# Weekly intravenous nanoparticle albumin-bound paclitaxel for elderly patients with stage IV non-small-cell lung cancer: a series of 20 cases

**DOI:** 10.7555/JBR.26.20110106

**Published:** 2012-05-20

**Authors:** Qi Zheng, Yu Yao, Kejun Nan

**Affiliations:** Department of Medical Oncology, the First Affiliated Hospital, Xi'an Jiaotong University, Xi'an, Shaanxi 710061, China.

**Keywords:** non-small-cell lung cancer, nanoparticles, albumin-bound, paclitaxel

## Abstract

The purpose of this study was to evaluate the efficacy and safety of nanoparticle albumin-bound paclitaxel as a rescue regimen in the treatment of patients with advanced non-small-cell lung cancer. We retrospectively reviewed the medical records of 20 patients with stage IV non-small-cell lung cancer. The patients had progressive disease after standard antitumor therapy and subsequently received intravenous albumin-bound paclitaxel at the dose of 100 mg/m^2^ in weekly schedule. Cumulative findings showed that the overall response rate was 30.0%, the disease control rate amounted to 40%, and the 1 year survival rate was 30%. In addition, the median time to progression and the median survival time reached 5 and 10 months, respectively. Meanwhile, no severe hypersensitivity reactions and grade 4 adverse effects were reported. In summary, weekly-administered albumin-bound paclitaxel seems to be an effective and safe regimen for elderly patients with stage IV non-small-cell lung cancer who were refractory to conventional therapy.

## INTRODUCTION

Lung cancer is the most common malignancy and the leading cause of cancer-related deaths worldwide, and is responsible for approximately 13% (1,608,800; 1,095,200 in males and 513,600 in females) of the total new cases and 18% (1,378,400; 951,000 in males and 427,400 in females) of the deaths per year[1]. The predominant form of this disease is non-small-cell lung cancer (NSCLC), which accounts for more than 80% of all cases of lung cancer[2] and remains incurable in the metastatic setting.

Paclitaxel, a hydrophobic chemotherapeutic agent, plays an important role in the treatment of advanced NSCLC[3],[4]. Use of this drug, however, is greatly limited by its solvent-based formulations relevant to Cremophor EL (CrEL), which produces serious and doselimiting toxicities. Administration of CrEL paclitaxel routinely requires long infusion duration (typically 3-24 h), special infusion sets (tubing and in-line filters), premedication with steroids and antihistamines to prevent hypersensitivity reactions[3]. Regardless of these precautions, severe and even fatal hypersensitivity still occurs occasionally[5]. In addition, CrEL can provoke hyperlipidemia, abnormal lipoprotein patterns, aggregation of erythrocytes, neutropenia, and prolonged and even irreversible sensory neuropathy[6]-[9]. In particular, CrEL negatively impacts on the efficacy of paclitaxel by forming micelles that entrap paclitaxel in the plasma compartment[10].

Nanoparticle albumin-bound paclitaxel (NAB-paclitaxel, ABI-007) is a Cremophor-free, 130-nanometer particle form of paclitaxel, which is designed to deliver paclitaxel as a suspension of albumin particles in saline. Because of the elimination of CrEL from its formulations, NAB-paclitaxel successfully minimizes the risk of hypersensitivity reactions, excludes CrEL-related toxicities, obviates the required steroid and antihistamine premedication and can be given over a shorter period without special infusion sets[11]. Notably, albumin has the natural ability to promote drug delivery to tumors by initiating albumin receptor (gp60)-mediated transcytosis across endothelial cells[12] and accumulating drug in tumors *via* binding to secreted protein acidic and rich in cysteine (SPARC)[13]. NAB-paclitaxel has been extensively studied as the first-line therapeutic agent aiming at advanced NSCLC, exhibiting unequivocal antitumor activity and minor side effects[14],[15]. Additional studies are ongoing to explore NAB-paclitaxel in combination with platinum-based regimens with and without bevacizumab as initial therapy in NSCLC[16],[17]. Nonetheless, little is known about the effect of NAB-paclitaxel as the second- or third-line therapy targeting NSCLC with advanced stage.

The aim of this retrospective study was to investigate the anticancer effect and toxicity of weekly administered NAB-paclitaxel as third-line chemotherapy in treating elderly patients with stage IV NSCLC who failed conventional standard therapy.

## SUBJECTS AND METHODS

### Subjects

Between January, 2010 and February, 2011, 20 patients received weekly NAB-paclitaxel treatment at the Department of Medical Oncology, the First Affiliated Hospital, Xi'an Jiaotong University. Patients were given pathological diagnoses of NSCLC. The clinical stage was determined on the basis of disease history, physical examination, systematic computed tomography (CT), bone scan, and magnetic resonance imaging of the brain. Prior use of taxanes (paclitaxel or docetaxel) and EGFR-TKIs [tyrosine kinase inhibitors of the epidermal growth factor receptor (EGFR); e.g. erlotinib or gefitinib] was permitted. Eastern Cooperative Oncology Group (ECOG) performance status (PS) of 0 to 2 was needed for inclusion in this study. Patients were excluded if they had symptomatic brain metastases, or a serious concurrent illness that was likely to weaken full compliance with the study, or a pre-existing peripheral neuropathy (grade 1 or higher), or any contraindication of chemotherapy. This study was approved by the Institutional Review Board of our hospital, and written informed consents were obtained from all patients before administration.

### Treatment

NAB-paclitaxel (Abraxanew; Abraxis Bioscience, Los Angels, CA, USA) was administered weekly on d 1, 8, and 15, followed by 1 week of rest. Doses were 100 mg/m^2^ administered as intravenous infusions over 30 min. Treatment was repeated every 4 weeks until disease progression or unacceptable toxicity happened. Premedication to prevent hypersensitivity reactions was not recommended. When patients underwent III/IV grade neutropenia or thrombocytopenia during the treatment, subcutaneous injection of granulocyte colony stimulating factor or interleukin-11 was recommended to address such hematological toxicities.

All patients had baseline CT examination of the chest and reassessment every two treatment cycles. Tumor responses were categorized based on the Response Evaluation Criteria in Solid Tumors (RECIST) standard[18]. Patients who finished more than one cycle of NAB-paclitaxel therapy were selected for toxicity analysis. Adverse events (AEs) were graded according to the National Cancer Institute's Common Toxicity Criteria (NCI CTC, Version 3.0).

## RESULTS

### Therapeutic outcome of NSCLC patients receiving nanoparticle-paclitaxel chemotherapy

The baseline characteristics of patients are presented in ***Table 1***. The group was composed of 11 males (55%) and 9 females (45%) who had a life expectancy of over 12 weeks and possessed adequate hematologic, hepatic, and renal function. The mean age was 66 years (60 to 76 years). Patients had previously undergone no less than 2 cycles of standard chemotherapy, but the disease progressed. Twenty patients completed at least two cycles of weekly NAB-paclitaxel chemotherapy, which added up to a total of 84 completed cycles.

**Fig. 1:**
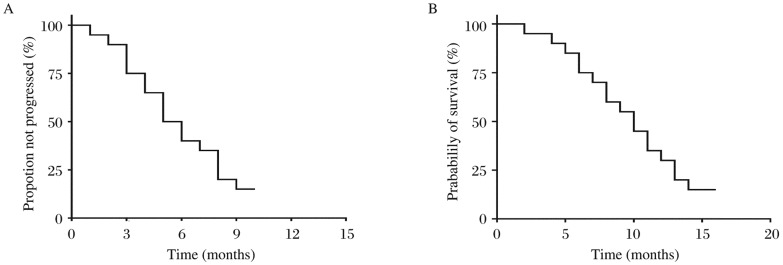
Time to disease progression (A) and overall survival (B) of subjects with non-small-cell lung cancer treated with nanoparticle albumin-bound paclitaxel.

Therapeutic effect was evaluated using RECIST standard: complete response (CR) 0 case, partial response (PR) 6 cases, stable disease (SD) 2 cases, progressive disease (PD) 12 cases, the overall response rate (ORR, CR+PR) was 30.0% (6/20; 95% CI=9%-49%) and the disease control rate (DCR, CR+PR+SD) was 40% (8/20; 95% CI=15%-59%). Subsequent follow-up showed that the median time to progression (TTP) was 5 months (95% CI=3.2-6.8 months; ***Fig. 1A***), the median survival time (MST) was 10 months (95% CI=7.8-12.2 months; ***Fig. 1B***), and the 1 year survival rate was 30% (6/20). As for 6 patients who had PR, all were diagnosed as adenocarcinoma, 3 of them had received docetaxel chemotherapy and others had a prior history of gefitinib or erlotinib. Meanwhile, 2 patients who had SD were both squamous carcinoma and received prior paclitaxel therapy.

**Table 1:** Baseline characteristics of patients treated with NAB-paclitaxel

Characteristic	Number of patients	Percentage(%)
Age(years old)		
≤65	6	30
> 65	14	70
Sex		
Female	9	45
Male	11	55
ECOG performance status		
0-1	14	70
2	6	30
Tumor pathological type		
Adenocarcinoma	8	40
Squamous carcinoma	12	60
Prior anti-tumor therapy		
Solvent-based paclitaxel	3	15
Docetaxel	5	25
Geftinib or erlotinib	6	30
Others	6	30
Smoking history		
Smoker	10	50
Non-smoker	10	50

(*n*=20)

**Table 2:** Toxicities of NAB-paclitaxel presenting as percentage of patients with adverse events

Toxicity	Maximum grade				
	All	1	2	3	4
Anemia	9(45)	5(25)	4(20)	0	0
Leukopenia	13(65)	4(20)	5(25)	4(20)	0
Neutropenia	12(60)	3(15)	5(25)	4(20)	0
Thrombocytopenia	2(10)	2(10)	0	0	0
Fatigue	14(70)	5(25)	6(30)	3(15)	0
Neuropathy	15(75)	7(35)	5(25)	3(15)	0
Alopecia	14(65)	7(30)	5(25)	2(10)	0
Constipation	12(60)	8(40)	4(20)	0	0
Rash	2(10)	1(5)	1(5)	0	0
Edema	3(15)	2(10)	1(5)	0	0
Myalgia	5(25)	4(20)	1(5)	0	0
Anorexia	6(30)	4(20)	2(10)	0	0
Hypersensitivity	0	0	0	0	0

[*n*(%)]

### Toxicity profile of NSCLC patients receiving nanoparticle-paclitaxel chemotherapy

On the whole, weekly NAB-paclitaxel at the dose of 100 mg/m^2^ was well tolerated, without treatment-related death or dose reduction. The median number of cycles administered was four. The most frequent AEs were those expected for paclitaxel in this patient population and are listed in ***Table 2***. No hypersensitivity reactions were observed. No episodes of treatment-related grade 4 AEs (including neutropenia) were reported. The major grade 3 toxicities were leukopenia (grade 3 in 20%), neutropenia (grade 3 in 15%), sensory neuropathy (grade 3 in 15%), fatigue (grade 3 in 15%) and alopecia (grade 3 in 10%). Other taxane-associated toxicities including anorexia, constipation, edema, myalgia and rash were mostly mild to moderate and easily managed.

## DISCUSSION

Although many therapeutic interventions have been developed to cope with NSCLC, the prognosis of advanced unresectable cancers remains dismal, and the 5-year survival rate for stage IV disease is almost 2%[19]. For stage IV NSCLC patients with a good ECOG PS, however, platinum-based chemotherapy is beneficial[20],[21]. Thus, platinum-based doublet regimens (e.g. paclitaxel with platinum) have been widely applied to the first-line treatment of patients with advanced NSCLC, which have yielded a 1-year survival rate of around 34%[22]. Furthermore, being superior to best supportive care with better survival and quality of life, docetaxel, pemetrexed and erlotinib or gefitinib are recommended as single agent second-line therapeutic regimens to counteract metastatic NSCLC[23]. When it comes to third-line therapy, up to now, there is no consensus for stage IV NSCLC. Though erlotinib has been used as a third-line regimen in patients with ECOG PS of 0-2, the ORR is only 8.9%, with an overall survival time of 6.7 months[24]. If disease progresses after the first and second-line therapies, patients are generally suggested to be treated with best supportive care or be enrolled in a clinical trial[23].

The salient point of our study is that single-agent NAB-paclitaxel had a favorable therapeutic effect on stage IV NSCLC. Among 20 patients who had PD after the conventional standard therapies, 8 (40%) responded to treatment with ABI-007, including 6 of PR and 2 of SD. The ORR and DCR was 30% and 40%, respectively, the median TTP was 5 months, the MST was 10 months, and the 1 year survival rate achieved 30%. In contrast, NAB-paclitaxel as first-line therapy of NSCLC at a dose of 260 mg/m^2^ every 3 weeks (q3w) yielded an ORR of 16%, a DCR of 49%, the median TTP of 6 months, the MST of 11 months, and the probability of surviving 1 year of 45%[14]. Another phase I/II trial of ABI-007 as initial chemotherapy at a dose of 125 mg/m^2^ in weekly schedule was conducted in elderly patients with stage IV NSCLC, and resulted in 1 year survival rate of 41%, ORR and DCR of 30% and 50%, respectively, with the median TTP and MST of 5 and 11 months, respectively[15].

Lung cancer is traditionally divided into two histological types: NSCLC (80.4%) and small cell lung cancer (SCLC) (16.8%)[25]. Moreover, the major forms of NSCLC, according to the classification of the World Health Organization (WHO), are squamous cell carcinoma, adenocarcinoma and large-cell (undifferentiated) carcinoma[26]. Histologically analyzing the 8 patients who benefited from ABI-007 in our study, 6 cases of PR had adenocarcinoma and the 2 cases of SD had squamous carcinoma, suggestive of the histologic predominance of adenocarcinoma. It is also reported that patients with adenocarcinoma receiving weekly NAB-paclitaxel had better efficacy in comparison with the q3w schedule, and the converse circumstance occurred in patients with squamous carcinoma[27]. Interestingly, antitumor responses were observed in 62.5% (5/8) patients previously treated with paclitaxel or docetaxel, in agreement with the results of two former clinical trials[28],[29]. Furthermore, 50% (3/6) patients with prior treatment with EGFR-TKIs achieved PR. NAB-paclitaxel, therefore, may serve as a rescue regimen for patients resistant to traditional taxanes or EGFR-TKIs.

The outstanding effectiveness of NAB-paclitaxel may be ascribed mainly to the promoted intratumor paclitaxel concentrations and the increased administered doses. An *in vivo* study proved that intratumor paclitaxel accumulation was 33% higher for NAB-paclitaxel than that for CrEL-paclitaxel when administered with equal doses of paclitaxel[30]. In another preclinical study using tumor-bearing mice[28] ABI-007 also showed 30% to 40% higher intratumor paclitaxel concentrations compared with equal doses of CrEL-paclitaxel. When utilized clinically, the equitoxic paclitaxel dose of ABI-007 was 50% to 70% higher than that of CrEL-paclitaxel[31]-[33]. On the other hand, both weekly and q3w regimens were effective in patients with advanced NSCLC, but the former seemingly brought out better clinical outcomes[27], and 100 mg/m^2^ had been determined to be the appropriate weekly dosage for heavily treated patients[28].

In terms of treatment-related toxicities, those of weekly NAB-paclitaxel were relatively mild and controllable, which was consistent with published literatures[14],[15]. In regard to hematological toxicity, no febrile neutropenia occurred, and the incidence of grade 2 and 3 neutropenia was 15% and 5%, respectively. Neither dose delays nor dose reductions were required owing to myelosuppression. The major nonhematologic toxicities were sensory neuropathy (75%) and fatigue (70%), but they were cumulative over cycles of therapy and typically improved with cessation of NAB-paclitaxel. As a matter of fact, the incidence of most toxicities was lower in the ABI-007 group than that of the CrEL paclitaxel group[31]-[33]. In contrast with the q3w schedule, additionally, weekly schedule of ABI-007 produced significant reductions in the incidence of peripheral neuropathy, myalgia, arthralgia, and alopecia[27].

As expected, NAB-paclitaxel treatment in weekly schedule was well performed and tolerated in elderly NSCLC patients (14/20 patients > 65 years old). In fact, over 50% of lung cancer patients are diagnosed at age older than 65 years and around 30% are diagnosed at age beyond 70 years[34],[35]. Generally speaking, ageing is associated with not only significant physiological changes (functional status, organ function, and drug pharmacokinetics, etc.), but also a series of comorbidities such as hypertension and diabetes, which inevitably affect functional status, general health and tumor symptoms of patients. What's more, hypertension and diabetes usually limit high dose of steroid in chemotherapy. On the other hand, weekly dosing NAB-paclitaxel was recently reported to have higher tumor response rate than q3w dosing in patients over 65 years old[36]. Considering the anticancer index and side effects, NAB-paclitaxel does gain an advantage over most of chemotherapeutic agents in aged patients. Weekly-administered Nab-paclitaxel, accordingly, seems to be an ideal regimen for elderly NSCLC patients, especially when use of steroid is contraindicated.

In conclusion, NAB-paclitaxel displays convincing antitumor activity in the management of stage IV NSCLC for elderly patients with refractory disease. With high therapeutic index and low side effects, weekly NAB-paclitaxel schedule seems to have the optimal clinical benefit-risk ratio in aged patients. Further prospective studies with large sample are warranted to explore the potential efficacy of NAB-paclitaxel in the treatment of advanced NSCLC.
